# Phase Angle Cut-Off Points and Their Association With Sarcopenia and Frailty in Adults of 50–64 Years Old and Older Adults in Mexico City

**DOI:** 10.3389/fmed.2021.617126

**Published:** 2021-03-15

**Authors:** Oscar Rosas-Carrasco, Roxana E. Ruiz-Valenzuela, Miriam T. López-Teros

**Affiliations:** ^1^Health Department, Ibero American University, Mexico City, Mexico; ^2^Instituto Nacional de Geriatría, Mexico City, Mexico

**Keywords:** phase angle, cut-off point, sarcopenia, frailty, older adults, sensitivity, specificity, validity

## Abstract

**Background:** In recent studies, the usefulness of the phase angle (PA) to assess geriatric conditions such as sarcopenia and frailty has been evaluated. However, there are no useful cut-off points for clinical research and/or practice.

**Objective:** To analyze PA cut-off points associated with sarcopenia and frailty in adults of 50-64 years old and older adults in Mexico City.

**Design:** Cross-sectional analysis of the FraDySMex cohort study (Frailty, Dynapenia, and Sarcopenia in Mexican Adults).

**Setting and Participants:** 498 people were included, 78.7% women, aged 71.1 ± 9.5 years. Methods: The sarcopenia measurements were made according to the European Working Group on Sarcopenia in Older People (EWGSOP) (2019) (by dynamometer to evaluate hand grip strength and dual energy X-ray absorptiometry (DXA) for appendicular muscle mass), and the frailty through the physical frailty phenotype with cut-off points adjusted to the Mexican population. The PA was evaluated by bioelectrical impedance analysis (BIA), tetrapolar to 50 Hz, other variables such as socio-demographic, comorbidity, cognitive status, and functional dependence were evaluated.

**Results:** The prevalence of frailty was 10.6% and sarcopenia 10.0%. The mean of the PA was 4.6° ± 0.70°. The PA cut-off point for frailty in adults 50 to 64 years was ≤4.3° [sensitivity (S) = 91.95%, specificity (Sp) 66.77%, AUROC (Area Under the Receiver Operating Characteristic) curve = 0.9273 95% CI (0.8720-0.9825)]; the PA cut-off point for sarcopenia was ≤4.3 [S = 91.95%, Sp = 66.77%, AUROC = 0.9306 95% CI (0.8508-1.000)]. The PA cut-off for frailty in adults ≥ 65 years was ≤4.1° [S = 72.37%, Sp 71.43%, AUROC = 0.7925 95%, CI (0.7280-0.8568)] for sarcopenia was ≤4.1° [S = 72.76%, Sp 73.81%, AUROC = 0.7930 95% CI (0.7272-0.8587)]. These cut-off points showed a significant association between PA with frailty (OR 4.84; 95% CI 2.61-8.99) and sarcopenia (OR 8.44; 95% CI 3.85-18.4) after adjusted by age, sex, BMI, comorbidity index and cognitive impairment.

**Conclusions and Implications:** These cut-off points of PA could be useful for the screening of sarcopenia and frailty in Mexican adults of 50 years and older in centers that have BIA.

## Introduction

The bioelectrical impedance analysis (BIA) is a relatively simple, inexpensive, fast, non-invasive, and reliable technique to assess body composition (1, 2). The BIA technique is based on the measurement of impedance made up of resistance (R) and reactance (Xc) through one or more electrical frequencies. The tangent area between resistance and reactance in a series or parallel circuit is called the phase angle (PA). The R and Xc values allow us to obtain, through various prediction equations, fat free mass (FFM), total body water (TBW), and fat mass (FM). The phase angle has its advantages, since it allows us to directly assess the permeability of the membrane by measuring intra and extracellular electrical flows, which makes it independent of the state of hydration, body weight, and does not require calculation using predictive models (3).

The PA represents an effective marker to preventively detect health conditions, as well as mortality, morbidity and lower survival with an established disease (4, 5). Due to its usefulness and simplicity, recent studies have explored PA cut-off points which can be effective in timely detecting conditions related to the functionality of the older adults, such as sarcopenia and frailty. Sarcopenia is the progressive and generalized loss of muscle mass and strength with the risk of adverse effects such as physical disability, poor quality of life and higher mortality (6). Frailty is the decrease in physiological reserve that would result in an increased risk of disability, loss of resistance and increased vulnerability to adverse events in individuals, which manifests itself in increased morbidity and mortality (7). According to a systematic review and meta-analysis, the prevalence of sarcopenia has been reported to be 10% for men (95% CI: 8-12%) and women (95% CI: 8-13%) respectively (8). According to a sample of adults over 60 years from Mexico City, the prevalence of sarcopenia was 9.7% and frailty was 15.7% (9, 10). Both conditions have serious clinical implications and, if detected in time, can be reversed with the support of an adequate treatment (11).

Recently the phase angle has been suggested as a possible effective biomarker for the prediction of both clinical conditions (5, 12). For example, Marini et al. (13) correlated a lower PA 5.2° in women and 5.0° in men with pre-sarcopenia defined by their muscle mass in their lower and upper extremities muscle mass <*7*.26 kg/m2 for men and 5.45 kg/m^2^ for women (14). Likewise, in a Japanese population of older adults who were hospitalized, Yamada et al. (15). found a PA of 4.05° and 3.55° in men and women, respectively, being effective indicators for muscle function (measured by ultrasonography for the quality of muscle mass, muscle strength, and physical performance). Other authors have reported different cut-off points of PA for sarcopenia: 4.55°, 5.6° for men and 5.8° for women (16, 17). The PA has also been correlated with muscular arm strength in people with cirrhosis (*r* = 0.53) and cancer (men *r* = 0.59, women *r* = 0.48) as well as with a knee extension (*r* = 0.4) (12, 18, 19). Similarly, a lower PA has been correlated with a greater degree of physical frailty, according to the Fried scale (*r* = −0.31) and ETF (Essential Frailty Toolset) (*r* = −0.31) (5).

Although it is true that low PA values have proven to be predictive of negative outcomes, there is currently a wide variability in reported cut-off points. This variability may depend on determinants of PA such as sex, age, BMI and the type of clinical condition or disease (18, 19). In addition, it is important to consider other parameters such as the type of population studied (hospital, community, homes for the elderly), among others. To our knowledge, there are no PA cut-off points for adults between 50 and 64 years old and older adults, adjusted by sex and BMI, related to health conditions that allow its use in different clinical and research settings. Therefore, the objective was to report the cut-off points associated with sarcopenia and frailty in adults of 50-64 years and Mexican older adults.

## Materials and Methods

### Design and Study Population

This study, a secondary analysis of the FraDySMex study (Frailty, Dynapenia and Sarcopenia in Mexican Adults), is a cohort of adults living in the community of two municipalities of Mexico City consisting of men and women over 50 years of age, all of whom are able to move with or without assistive devices and able to answer the questions of the study questionnaire by themselves or with the help of a caregiver if the score of the mini-mental state examination (MMSE) with 10 points or less. People with a total functional dependence, presence of edema in their extremities, current intake of diuretics, presence of fever, diarrhea, pacemaker carriers, cancer diagnosis of 5 years or less, were excluded. The study consisted of objective evaluations by the multidisciplinary team of the Research Laboratory in Functional Evaluation of the National Institute of Geriatrics in Mexico City. More details of the design, recruitment and selection of the FraDySMex study of participants can be found in another study (20). The study was approved by the Ethics Committee of the Mocel de Angeles General Hospital and enrolled in the National Institute of Geriatrics with the number DI-PI-002/2014. Informed signed consent was obtained by all individuals before the study.

### Measurements

Phase angle. It was evaluated by the 50 Hz frequency bioelectrical impedance tetrapolar, brand SECA® model mBCA 514.

### Diagnosis of Sarcopenia and Frailty

Sarcopenia was defined according to the criteria of the EWGSOP 2019 (6) adjusted to our population considering the low muscle strength (criterion 1), low muscle quantity (criterion 2) and the low physical performance (criterion 3). Probable sarcopenia is identified by criterion 1, diagnosis is confirmed by additional documentation of criterion 1 and 2 and criteria 1, 2, and 3 are all met, sarcopenia is considered severe. For this analysis, it was classified as sarcopenic when had sarcopenia confirmed and sarcopenia severe and as non-sarcopenic when had sarcopenia probable or don't present any criterion.

Muscle strength was evaluated with manual hand grip strength using the hydraulic JAMAR dynamometer, Lafayette, IN. Three measures of the dominant hand were taken and the highest was considered for the analysis. For low muscle strength, the lowest quartile for grip strength (kg) was considered adjusted for BMI (kg / mts^2^) and sex (Table 1). Gait speed (GS) was recorded at a habitual gait of 6 meters on a Gait Rite instrumented mat (platinum 20) (204 x 35.5 x 0.25 inches, 100 Hz sampling rate). The GS cut-off points for our population were adjusted for height (m) and sex based on the lowest quintile (Table 1). Muscle quantity was evaluated with appendicular muscle mass (ASM) through the dual energy X-ray absorptiometry (DXA), (Hologic Discovery-WI; Hologic Inc., Bedford-MA; to define low muscle mass based on the lowest quintile for sex (Table 1).

**Table 1:** Components and cut-off points used for the diagnosis of sarcopenia.

**Sex**	**ASM^a^**	**Gait speed^b^**	**Hand-grip strength^c^**
Males	ASM ≤ 6.68 kg/m^2^	Height ≤ 1.65m ≥ 5.7 s Height > 1.65m ≥4.5 s	BMI ≤24.3 kg/m^2^ ≤22 Kg BMI 24.4-26.6 kg/m^2^ ≤22 Kg BMI 26.7-28.5 kg/m^2^ ≤24 Kg BMI >28.5 kg/m^2^ ≤22 Kg
Females	ASM ≤ 5.35 kg/m^2^	Height ≤ 1.51m ≥ 6.8 s Height > 1.51m ≥5.4 s	BMI ≤ 24.7 kg/m^2^ ≤12 Kg BMI 24.8-27.6 kg/m^2^ ≤12 Kg BMI 27.7-30.5 kg/m^2^ ≤12 Kg BMI >30.5 kg/m^2^ ≤13 Kg

*ASM, Appendicular muscle mass*.

a *Cut-off points according to the lowest quintile of ASM*.

b *Cut-off points by height according to the lowest quintile of gait speed*.

c *Cut-off points by BMI quartile*.

### Physical Frailty

Using the Fried's criteria a score ≥ 3 was consider as frailty (21). The grip strength and gait velocity were defined as described in the sarcopenia variable. Low physical activity was defined using the lowest quintile of kilocalories per week obtained through the physical activity questionnaire for older adults (CHAMPS), <545.7 for men and <481.2 kcal/week for women (22). The following question was used for the variables of involuntary weight loss: In the last year, have you unintentionally lost 5 kg (or 5% of your weight) or more? For the low energy or exhaustion variable, 2 questions of the CES D-7 scale Mexican version were used (does it feel like everything you do is an effort? and the one that questioned if the person felt like doing nothing. These questions were answered as never or almost never, sometimes (1 to 2 times a week), frequently (3 to 4 days a week) and 1 (always or almost always (5 to 7 days a week) (23).

### Other Variables

Other measures obtained were the following: depressive symptoms using the CES scale item D-7 (Center for Epidemiologic Studies, Depression Scale, Mexican version) (depression was considered if it scored ≥5) (23). Cognitive state was assessed using the MMSE (cognitive impairment was considered when it scored ≤ 23 points with ≤ 5 of school education, ≤ 19 points he/she was in school between 1 and 4 years, ≤ 16 without schooling or <1 year of schooling) (24, 25). Comorbidity was assessed using the comorbidity index adapted to Mexican Spanish (26, 27). Information about schooling in years (<10 y vs. ≥ 10 y), history of falls (one or more falls in the last year), low physical performance was assessed by the short physical performance battery (SPPB) using ≤ 8 points as the cut-off point (28). Functional dependence was also assessed using the Lawton scale for instrumental activities of daily life (IADL) (≥ 1 activities) and the Barthel scale for basic activities of daily living (BADL) (≤95 points) (29, 30). Malnutrition was evaluated with Mini Nutritional Assessment (MNA) scale and cutoff ≤ 23 points was used to define the risk of malnutrition. Other measures of body composition were also obtained through DXA, such as the percent total body fat considering obesity when calculated as > 30% in men and > 40% in women, bone mineral density of the hip and spine using the WHO cut-off points to define osteopenia and osteoporosis (31). Similarly, anthropometric measurements were obtained such as weight (kg), height (mts), calf and mild arm circumference (cm), and BMI (kg / mts^2^).

### Statistical Analysis

The data were analyzed using the Stata 12.0 statistical package. Descriptive statistics are reported as means ± SD for continuous variables and as frequencies for categorical variables. Some continuous variables were dichotomized for its analysis according to cut-off points previously established in the literature as exposed in the variable section. Determination of the cut-off points of the PA. The cut-off points were explored by sarcopenia and frailty in both aged groups) using sensitivity (S), specificity (Sp), and AUROC curve analysis.

To show the association of these cut-off points of PA with sarcopenia and frailty, were included in a simple and adjusted logistical regression. The results are shown as odds ratio (OR) with the respective 95% confidence intervals (CI); the final model was adjusted by sex and other variables reported in the literature that are associated with phase angle, sarcopenia, and frailty. To evaluate the goodness of fit of the models, we use Hosmer-Lemeshow goodness of fit test and AUCROC curve. The interaction and collinearity between the independent variables of the model were also evaluated.

## Results

**Figure 1:**
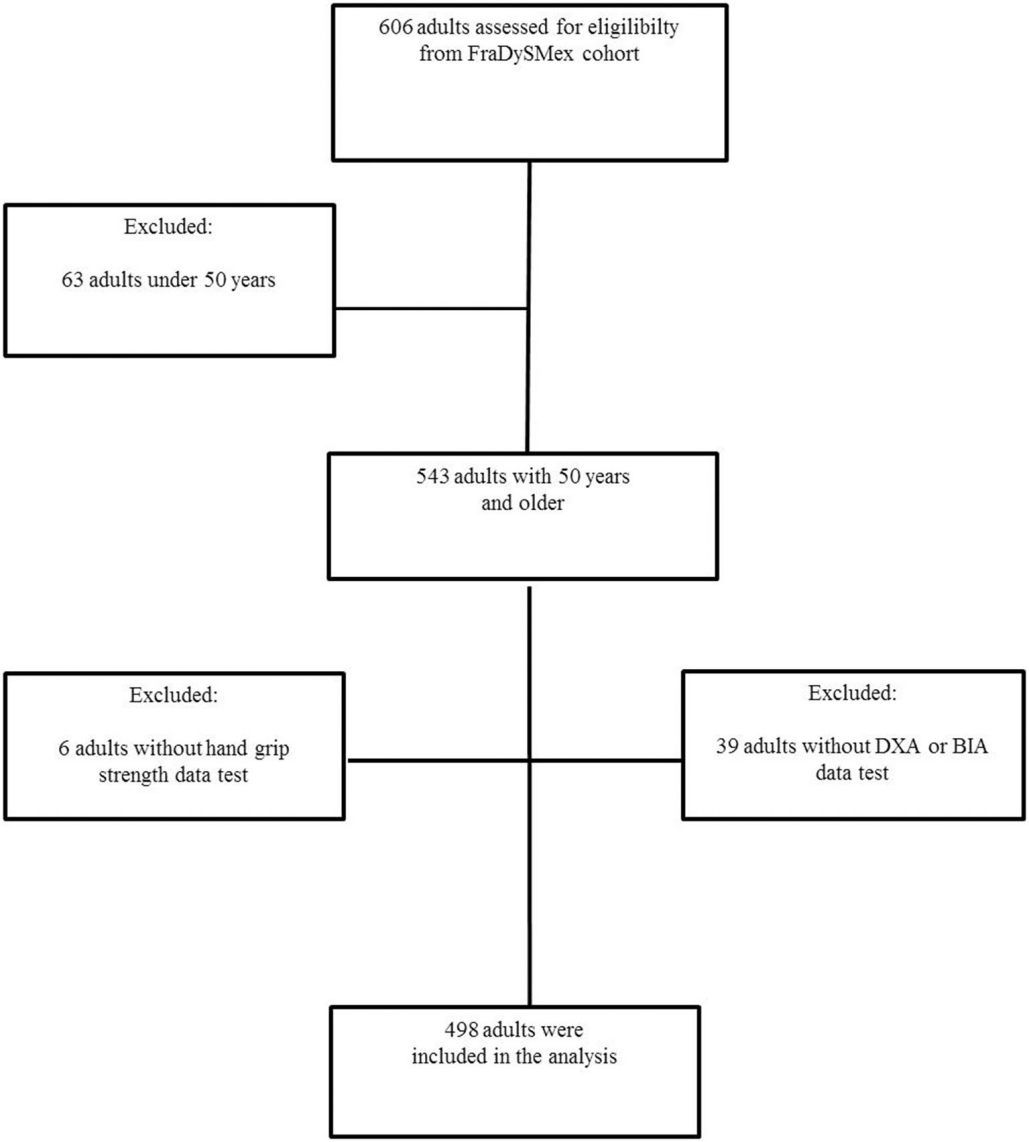
Flowchart of the FraDySMex study (Frailty, Dynapenia and Sarcopenia in Mexican Adults). BIA (bioelectrical impedance) and DXA (dual energy X-ray absorptiometry).

The analytical sample consisted of 606 after excluding 63 participants younger than 50 years, 39 participants not submitted to DXA or BIA evaluation and 6 participants not submitted to hand-grip test (Figure 1). 498 adults over 50 years old were included, with 78.7% females (*n* = 392); the mean age was 71.1 ± 9.5 (SD) years and 50.3% had low schooling. The following prevalence's were observed in the total sample: depressive symptoms (41.8%), cognitive impairment (11.2%), higher comorbidity (24.9%), falls (40.9%), low physical performance (34%), functional dependence by IADL (16.4%) and BADL (8.0%), risk of malnutrition (24.6%), osteopenia/osteoporosis (47.0%), and obesity (70.1%). A prevalence of frailty of 10.6% and sarcopenia of 10.0% were found in the total sample (Table 2). The average of the PA in the total adults was 4.6° ± 0.70° (SD) and 5.0 ± 0.61 in the younger adults (50 to 65 years) and 4.3 ± 0.70 in the older adults (over 65 years), *p* = 0.0000. The PA cut-off points were generated for the two conditions (frailty and sarcopenia) stratified by age (adults with 50 to 65 years and adults over 65 years) reported (Table 2); the PA cut-off for frailty in adults 50 to 65 years was ≤4.3°, S = 91.95%, Sp 66.77% LHR (likelihood ratio) (+) 2.7584, LHR (-) 0.1208; AUROC = 0.9273 95% CI (0.8720-0.9825) (Table 3). PA cut-off for sarcopenia in the adults 50 to 65 years was ≤4.3°, S = 91.95%, Sp 66.77% LHR (+) 2.7589, LHR (-) 0.1208; AUROC = 0.9306 95% CI (0.8508-1.000) (Table 3). The PA cut off for frailty in adults over 65 years was ≤4.1°, S = 72.37%, Sp71.43% LHR (+) 2.5329, LHR (-) 0.3868; AUROC = 0.7925 95% CI (0.7280-0.8568). PA cut-off for sarcopenia in adults over 65 years was ≤4.1°, S = 72.76%, Sp= 73.81% LHR (+) 2.7788, LHR (-) 0.3691; AUROC = 0.7930 95% CI (0.7272-0.8587) (Table 3).

**Table 2:** General characteristics (*n* = 498).

**Characteristics**	**Total**	**50-65, years (*n* = 152)**	**> 65 years (*n* = 346)**	***P***
	**Mean ± SD or *n* (%)**			
Age, years	71.1 ± 9.5	59.7 ± 4.5	76.1 ± 6.4	0.0000
Sex;				0.2132
Women	392 (78.7)	123 (31.4)	269 (68.6)	
Men	106 (21.2)	29 (27.4)	77 (72.6)	
Schooling ≤ 9 years	250 (50.3)	55 (22.0)	195 (78.0)	0.0000
Cognitive impairment (MMSE adjusted for schooling)	56 (11.2)	10 (17.9)	46 (82.1)	0.0289
Depressive symptoms (CESD-7 ≥5 points)	231 (41.8)	48 (28.24)	122 (71.8)	0.4260
Comorbidity Index (Charlson scale ≥ 3 points)	124 (24.9)	32 (25.8)	92 (74.2)	0.4260
Falls (≥1 fall in the last year)	204 (40.9)	60 (29-4)	144 (70.6)	0.2070
Malnutrition (MNA scale ≤23 points)	136(24.6)	37 (27.2)	99 (72.8)	0.1489
BADL (Barthel index ≤90 total score)	40 (8.0)	5 (12.5)	35 (87.5)	0.0098
IADL (Lawton ≥1 activities)	82 (16.4)	7 (8.5)	75 (91.5)	0.0000
Low physical performance (SPPB scale ≤ 8 points	169 (34)	22 (13.0)	147 (86.9)	0.0000
Frailty (Fried Phenotype≥ 3 total score)	53 (10.6)	16 (30.1)	37 (69.8)	0.0003
Sarcopenia (EWGSOP, 2019)	50 (10.0)	4 (8.0)	46 (9.0)	0.0001
Phase angle (°)	4.6 ± 0.70	5.0 ± 0.61	4.3 ± 0.70	0.0000
Calf circumference, (cm)	34.7 ± 4.8	35.7 ± 5.2	34.3 ± 4.7	0.0050
Mild arm circumference, (cm)	29.9 ± 3.9	31.1 ± 3.8	39-5 ± 3.9	0.0000
Weight (kg)	65.8 ± 12.6	68.2 ± 12.8	65.2 ± 12.2	0.0138
Height (m)	1.53 ± 0.09	1.54 ± 0.09	1.52 ± 0.09	0.0014
BMI (kg/m^2^)	27.9 ± 4.7	28.5 ± 4.9	28.3 ± 4.6	0.5226
Total lean mass (kg)	36.5 ± 7.7	37.6 ± 7.8	36.0 ± 7.7	0.0376
ASM/ht2 (kg/m^2^)	6.4 ± 1.1	6.4 ± 1.1	6.3 ± 1.1	0.2737
Obesity, %	378 (70.1)	124 (32.8)	254 (67.2)	0.4098
Osteopenia/osteoporosis	252 (47.0)	52 (20.6)	200 (79.4)	0.0005
Hand-grip strength, (kg)	16.7 ± 7.2	19.1 ± 7.3	15.6 ± 7.1	0.0000
Gait speed, (seconds)	5.3 ± 2.8	4.3 ± 0.95	5.7 ± 3.2	0.0000

*Data presented as mean (standard deviation) or number (%). ADL, activities of daily living; ASM/ht2, appendicular skeletal muscle mass adjusted by height square; BMI, body mass index; EWGSOP, European Working Group on Sarcopenia in Older People; IADL, instrumental activities of daily living; MMSE, the Mini-Mental State Examination, MNA (mini nutritional assessment), CES-D (Center for Epidemiologic Studies, Depression Scale), SPPB (short physical performance battery)*.

*This table presents the general characteristics that describe the study population*.

**Table 3:** Criterion validity of phase angle vs. frailty and sarcopenia stratified by age.

**PA values**	**Sensitivity**	**Specificity**	**LHR+**	**LHR-**
50–65 years				
Frailty				
≤ 3.9	97.32%	0.00%	0.9732	——
≤ 4	96.64%	0.00%	0.9664	——
≤ 4.1	95.97%	33.33%	1.4396	0.1218
≤ 4.2	93.96%	33.33%	1.4094	0.1812
**≤ 4.3**	**91.95%**	**66.67%**	**2.7584**	**0.1208**
≤ 4.4	89.26%	66.67%	2.6779	0.1611
≤ 4.5	88.59%	100.0%	——	0.1141
≤ 4.6	85.23%	100.0%	——	0.1467
> 65 years				
Frailty				
≤ 3.9	83.55%	52.38%	1.7546	0.3140
≤ 4.0	78.95%	59.52%	1.9505	0.3537
**≤ 4.1**	**72.37%**	**71.43%**	**2.5329**	**0.3868**
≤ 4.2	66.12%	76.19%	2.7770	0.4447
≤ 4.3	59.21%	83.33%	3.5526	0.4895
≤ 4.4	53.95%	88.10%	4.5316	0.5228
≤ 4.5	44.08%	92.86%	6.1710	0.6022
≤ 4.6	36.51%	97.62%	15.3355	0.6504
50-65 years				
Sarcopenia				
≤ 3.9	97.99%	33.33%	1.4698	0.0604
≤ 4	97.32%	33.33%	1.4597	0.0805
≤ 4.1	95.97%	33.33%	1.4396	0.1208
≤ 4.2	93.96%	33.33%	1.4094	0.1812
**≤ 4.3**	**91.95%**	**66.67%**	**2.7584**	**0.1208**
≤ 4.4	89.26%	66.67%	2.6779	0.1611
≤ 4.5	87.92%	66.67%	2.6376	0.1812
≤ 4.6	85.23%	100.00%	——	0.1477
>65 years				
Sarcopenia				
≤ 3.9	84.39%	59.52%	2.0848	0.2663
≤ 4	79.40%	61.90%	2.0843	0.3327
**≤ 4.1**	**72.76%**	**73.81%**	**2.7788**	**0.3691**
≤ 4.2	66.45%	78.57%	3.1008	0.4721
≤ 4.3	59.47%	85.71%	4.1628	0.4729
≤ 4.4	53.49%	85.71%	3.7442	0.5426
≤ 4.5	43.85%	90.48%	4.6047	0.6206
≤ 4.6	36.54%	95.24%	7.6744	0.6666

*LHR, likelihood ratio; PA, phase angle. This table showed the PA cut-off points (in bold) and their association with sarcopenia and frailty, as well as their sensitivity, specificity and LHR*.

The Table 4 included two models, the first adjusted model show a significant association between the low phase angle cut-off point ≤ 4.3 in the adults between 50 to 65 years old and ≤4.1 in the adults over 65 years) and frailty (OR 4.84; 95% CI 2.61-8.99); in the second model show a significant association with sarcopenia (OR 8.44; 95% CI 3.85-18.4); both models were adjusted by sex, BMI, comorbidity index and cognitive impairment.

**Table 4:** Association between phase angle and sarcopenia and frailty.

	**Model with frailty phenotype**		**Model with sarcopenia**	
	**OR (95% CI), *p* crude**	**OR (95% CI), *p* adjusted**	**OR (95% CI), *p* crude**	**OR (95% CI), *p* adjusted**
Sex (women)	3.42 (1.20–9.70), 0.020	4.51 (1.14–16.63), 0.023	1.16 (0.53–2.54), 0.694	1.24 (0.47–3.26), 0.649
Comorbidity index	1.32 (0.710–2.45), 0.380	1.18 (0.57–2.47), 0.651	1.46(0.698–3.07), 0.312	1.31 (0.50–3.45), 0.571
Cognitive impairment	4.69 (2.47–8.904), 0.000	2.68 (1.14–6.29), 0.023	3.72 (1.71–8.09), 0.001	2.35 (0.87–6.32), 0.088
BMI	0.88 (0.63–1.22), 0.454	1.08 (0.76–1.54), 0.655	0.21 (0.12–0.34), 0.000	0.27 (0.17–0.46), 0.000
Low phase angle	5.51 (3.05–9.95), 0.000	4.84 (2.61–8.99) 0.000	12.79 (6.36–25.69), 0.000	8.44 (3.85–18.4), 0.021
Hosmer-Lemeshow	———	0.9945	———	0.7877
Goodness of fit				
AUROC of model	———	0.7852	———	0.8887

*AUROC area under the receiver operating characteristics; BMI body mass index; CI, confidence interval; OR, odds ratio. BMI was categorized in men as “0” <24.9, ^..^1^..^ 24.9-27.19, ^..^2^..^ >27.19, women “0” 25.19, ^..^1^..^25.19-28.11, ^..^2^..^>28.11. Low phase angle was considered as ≤4.3 in the adults with 50–65 years and ≤4.1 in the adults ≥65 years*.

## Discussion

Our results show two new PA cut-off points for sarcopenia, in the adults 50 to 65 years was ≤4.3° with a high S (91.95%) and AUROC = 0.9306 95% CI (0.8508-1.000) both low Sp (66.77%). In adults over 65 years was ≤4.1° with acceptable S = 72.76% and Sp 73.81% and AUROC = 0.7930 95% CI (0.7272-0.8587) both cut-off points can be used for the screening of sarcopenia; in this regard a few studies have been conducted on the relationship between PA and sarcopenia. In the study of Basile et al. (12), with 207 people (mean age 76.2 ± 6.7 years) it was shown that there is an inverse correlation between the muscle mass (y = 3.16 + 0.08x; *r* = 0.49; *P* < 0.001) and muscular strength (y = 3.04 + 0.25x; *r* = 0.60; *P* < 0001) with the PA without specifying cut-off points. Previously Kilic et al. (16), showed that a cut-off point of <4.55° with an AUROC of 0.703 (*P* < 0.0001), with a sensitivity of 70% and specificity of 64.9% were acceptable for screening sarcopenia. However, in other study by Santana et al. (17), with 146 hospitalized people (age 71.6 ± 7.6 years) did not find an association between PA values and sarcopenia components. The most recent study to date on this association by Pessoa et al. (32) with 94 women, did not find an association with sarcopenia OR = 1.50 (0.520–4.319), low muscle mass index OR = 1.50 (0.520–4.319), low HGS OR = 3.15 0.954–10.401). This study mentions that the small sample size could impact the lack of association in PA and sarcopenia.

Our study proposes these cut-off points (≤4.3° for adults 50 to 65 years and ≤4.1° for adults over 65 years) based on their criterion validity through the following properties: sensitivity, specificity, AUROC and LHR +, LHR-. However, to strengthen this criterion validity, the low PA variable was included as independent variable in a model adjusted to sarcopenia and its was associated with a OR = 8.44 (95% CI 3.85-18.4), *P* = 0.021, which demonstrates that these cut-off points remains associated after the adjustment with variables such as age, sex, cognitive impairment and comorbidity. Indeed, there are conflicting results on the association between PA and sarcopenia which can be explained by the diversity of the criteria used to define sarcopenia and the diversity of the population studied (older adults in the community, such as the population we used in this work, hospitalized elders, adults with some advanced disease such as kidney failure, liver cirrhosis, heart failure, among others).

The gait speed and muscle strength are two dimensions of physical frailty suggested by Fried et al. (6), which are closely related to sarcopenia. Our results show that cut-off points of ≤4.3° for adults 50 to 65 years and ≤4.1° for adults over 65 years are associated with frailty with an S = 91.95%, specificity 66.77%; AUROC = 0.9273 95% CI (0.8720-0.9825) and sensitivity = 72.37%, Sp= 71.43%; AUROC = 0.7925 95% CI (0.7280-0.8568), respectively by age group. In the model adjusted for age, sex, BMI, cognitive status and comorbidity, these cut-off points remained associated with an OR = 4.84 (2.61-8.99) *P* = 0.000, which shows that low PA is also associated with physical frailty and could be used for screening frailty when a BIA is available and a dynamometer is not. In this regard, the study by Mullie et al. (5) found a low PA (<4.5°, based on the first tertile of the population), has a high predictive capacity for postoperative mortality at one month, with an OR = 3.57 (1.35-9.47 95% CI) for each decrease of a PA degree. In another study of 4,667 people aged 60 years and over (4), in a low PA (first quintile) in women, the range of the first quintile was 2,655 to 5,419°, with a significant association, with an OR = 4.4 (95% CI 2.6−7-7), and in men the range of the first quintile of the PA was 3,070 to 5,646° with a significant association with an OR = 3.1 (95% CI 1.2-7-9). In this same study at 12 years of follow-up, low PA was associated with an HR = 2.4 (95% CI [95% CI] 1.8–3.1) in women and an HR = 2.2 (95% CI 1.7–2.9) in men, demonstrating the predictive capacity for frailty and mortality. The issue that makes it difficult to take the first quintile by sex is that different cut-off points must be taken into account for sex, age, among other variables, unlike in our article that proposes a single cut-off point adjusted by these same variables, which in the adjusted model remains significantly associated.

Some limitations were considered for this study; it is a cross-sectional study that does not allow assessing the temporality of the presentation of the variables, as well as limiting their predictive capacity. However, this study incorporates the new European criteria for sarcopenia adjusted to the Mexican population that could not be very comparable with previous studies because they included criteria of physical frailty of Fried et al. (6). However, an strength was to use the objective measures such as appendicular mass by DXA, gait speed by gait Rite® and hand grip strength by manual dynamometer (JAMAR®) to evaluate the main variables.

The cut-off points shown are not representative of the national context; however, since we do not have previous studies in this population, we believe that reporting a cut-off point of PA associated with sarcopenia and frailty contributes to establishing a criterion that can be used when the bioelectrical impedance is accessible (hospitals, nutricionist, medical and geriatrician offices).

## Conclusion and Implications

A PA with a cut-off point of ≤4.3 in the adults 50 to 65 years and ≤4.1° in adults over 65 years, showed association and acceptable sensitivity for the screening for sarcopenia and frailty in men and women. The PA can be indicator effective in timely detecting conditions related to the functionality of the older adults, such as sarcopenia and frailty. It is important to evaluate these geriatric conditions because are they associated with a greater functional dependence, institutionalization, higher health costs, and mortality.

## Data Availability Statement

The raw data supporting the conclusions of this article will be made available by the authors, without undue reservation.

## Ethics Statement

The studies involving human participants were reviewed and approved by Ethics Committee of the National Institute of Geriatrics with the number DI-PI-002/2014. The patients/participants provided their written informed consent to participate in this study.

## Author Contributions

ML-T and OR-C contributed to the data collection, original idea, data analysis, manuscript writing and revision. RR-V manuscript writing and revision. All authors contributed to the article and approved the submitted version.

## Conflict of Interest

The authors declare that the research was conducted in the absence of any commercial or financial relationships that could be construed as a potential conflict of interest.
